# Differential and sequential immunomodulatory role of neutrophils and Ly6C^hi^ inflammatory monocytes during antiviral antibody therapy

**DOI:** 10.1080/22221751.2021.1913068

**Published:** 2021-05-21

**Authors:** Jennifer Lambour, Mar Naranjo-Gomez, Myriam Boyer-Clavel, Mireia Pelegrin

**Affiliations:** a IGMM, Univ Montpellier, CNRS, Montpellier, France; b IRMB, Univ Montpellier, INSERM, CNRS, Montpellier, France; c Montpellier Ressources Imagerie, Biocampus, Univ Montpellier, CNRS, Montpellier, France

**Keywords:** Antiviral immune responses, monoclonal antibodies, immunotherapy, vaccinal effects, immune complexes, neutrophils, monocytes, FcγR

## Abstract

Antiviral monoclonal antibodies (mAbs) can generate protective immunity through Fc-FcγRs interactions. We previously showed a role for immune complexes (ICs) in the enhancement of antiviral T-cell responses through FcγR-mediated activation of dendritic cells (DCs). Here we addressed how mAb therapy in retrovirus-infected mice affects the activation of neutrophils and inflammatory monocytes, two FcγR-expressing innate effector cells rapidly recruited to sites of infection. We found that both cell-types activated *in vitro* by viral ICs secreted chemokines able to recruit monocytes and neutrophils themselves. Moreover, inflammatory cytokines potentiated chemokines and cytokines release by IC-activated cells and induced FcγRIV upregulation. Similarly, infection and mAb-treatment upregulated FcγRIV on neutrophils and inflammatory monocytes and enhanced their cytokines/chemokines secretion. Notably, upon antibody therapy neutrophils and inflammatory monocytes displayed distinct functional activation states and sequentially modulated the antiviral immune response by secreting Th1-type polarizing cytokines and chemokines, which occurred in a FcγRIV-dependent manner. Consistently, FcγRIV- blocking in mAb-treated, infected mice led to reduced immune protection. Our work provides new findings on the immunomodulatory role of neutrophils and monocytes in the enhancement of immune responses upon antiviral mAb therapy.

## Introduction

The development of powerful antiviral monoclonal antibodies (mAbs) has provided new therapeutic opportunities to treat severe viral infections, including emerging viral infections that threat global public health [1,2]. Fc-dependent mechanisms are crucial for efficient antiviral activity of neutralizing mAbs through the engagement of IgG receptors (FcγRs) expressed on immune cells. These Fc-FcγR interactions lead to the elimination of viral particles and virus-infected cells through phagocytic and cytotoxic mechanisms (i.e. antibody-dependent cellular phagocytosis (ADCP), antibody-dependent cell-mediated cytotoxicity (ADCC), …) [3]. Moreover, studies in different animal models of viral infection, including ours, have provided evidence that mAbs can also enhance antiviral immune responses (so called “vaccinal effects”) in a Fc-dependent manner [4]. These vaccinal effects have been recently reported in HIV-infected patients treated with broadly neutralizing mAb (bnAbs) [5–7] although the mechanisms involved have not been identified thus far. The elucidation of the molecular and cellular mechanisms driving Fc-dependent, mAb-mediated immunomodulation is therefore an important issue that will be key to achieving protective immunity against severe viral infections by mAbs.

While several Fc-mediated effector functions (i.e. ADCC, ADCP, … .) have been shown to be required for antibody-mediated antiviral protection [8–11], whether and how FcγR engagement by antiviral mAbs affects the immunomodulatory properties of different FcγR-expressing cells (i.e. cytokines/chemokines secretion, activation markers expression, …) has been little studied. In addition, the specific contribution of different FcγRs-expressing cells in the induction of vaccinal effects by mAbs still remains ill-understood. Multiple restrictions (i.e. technical and ethical issues, costs, …) largely limit those studies in humans and non-human primates (NHP). As an alternative, *in vivo* studies in immunocompetent mice infected with the Murine Leukemia Virus FrCasE allowed the identification of several immunological mechanisms that drive protective immunity upon mAb therapy [4,12]. We showed that treatment of FrCasE-infected mice with the neutralizing mAb 667 elicits protective adaptive antiviral immunity through the engagement of FcγRs [13,14]. Notably, mAbs form immune complexes (ICs) with viral determinants that enhance antiviral T-cell responses through FcγR-mediated binding to dendritic cells (DCs) [13,15–17]. Furthermore, we showed a key immunomodulatory role of neutrophils in the induction of protective humoral responses *via* the acquisition of B-cell helper functions (i.e. B-cell activating factor secretion) upon FcγR-triggering by the therapeutic mAb [18]. While the role of IC-activated DCs in the enhancement of antiviral immune responses has been addressed in several studies [12,19,20], the role of IC-activated neutrophils has mostly been overlooked. Evidence shows that, in addition to being key effector cells to fight against invading pathogens, neutrophils are also endowed with immunomodulatory properties through the secretion of a plethora of chemokines and cytokines [21–23]. Yet, the functional activation of neutrophils by viral ICs and the resulting effect on their immunomodulatory properties have poorly been studied in the context of antiviral mAbs therapies. Similar to neutrophils, inflammatory Ly6C^hi^ monocytes are also rapidly recruited to sites of infection and are key players to control viral spread [24]. In the context of antibody therapy, such viral propagation control by monocytes involves Fc-FcγR interactions [10]. However, the potential contribution of monocytes to the induction of vaccinal effects by antiviral mAb has not been reported thus far. As both neutrophils and inflammatory monocytes display multiple immunomodulatory functions and can mediate protective immunity, immunosuppression or immunopathology (i.e. in SARS-CoV2 infection) in a context dependent manner, it is important to dissect how antiviral mAb therapy shapes the phenotype and functional properties of these FcγR-expressing cells. Thus, a better understanding of IC-FcγR interactions on neutrophils and monocytes can not only help to improve immunotherapies for chronic and emerging viral infections but also answer fundamental questions related to antibody-mediated immunopathology.

Here, we report that neutrophils and monocytes activated *in vitro* by viral determinants secrete high levels of monocyte- and neutrophil-recruiting chemokines. *In vivo*, we have shown that viral infection and mAb-treatment shape the immunomodulatory properties of neutrophils and inflammatory monocytes. Our data show that the functional activation of both cell types differs in terms of cytokine and chemokine secretion, evolves overtime and is different in the presence or in the absence of mAb-treatment. Importantly, antibody therapy leads to increased secretion of proinflammatory cytokines and chemokines that are potent inducers of Th1-biased immune responses. These findings might help to improve mAb-based antiviral therapies by tailoring therapeutic interventions aiming at harnessing the immunomodulatory properties of these cells.

## Materials and methods

### Ethics statement

All experimental procedures were performed in accordance with the French national animal care guidelines (CEEA-LR-12147 approval, date of approval 14th May 2013, and 2018101018119517 #15305 v1 approval, date of approval 27th February 2019).

### Mice

Inbred 129/Sv/Ev mice (H-2D^b^ haplotype) were used and maintained under conventional, pathogen-free facilities at the Institut de Génétique Moléculaire de Montpellier (RAM-ZEFI). They have been used without distinction as to sex and at different ages according to experiments.

### Viral stocks

FrCasE [25] viral stocks were produced and stored as previously described in [26].

### Viral infection and immunotherapy

Eight-day-old 129/Sv/Ev mice were infected by intraperitoneal (i.p.) administration with 50 μl of a viral suspension containing 50,000 focus-forming units (FFU) and treated, or not, with 30 μg of 667 mAb targeting gp70 protein of viral envelope [27], 1-hour p.i. and on days 2 and 5 p.i. by i.p. administration. Mice were euthanized and spleens collected at days 8 and 14 p.i. The 667 mAb (kindly provided by Dr. John Portis, NIAID, Laboratory of Persistent Viral Diseases) was produced by BioXcell.

### Phenotypical and functional activation of FcγRIV-expressing cell from spleen ex vivo

Single-cell suspensions of splenocytes were obtained from naive, infected/non-treated and infected/treated mice at 8 and 14 days p.i. Spleen cell suspensions were obtained by mechanical dissociation in PBS, then filtered in 0,70 µm strainer. 20% of each spleen was used for immunophenotyping by FACS, 80% left was dedicated to neutrophils and monocytes sorting. Red blood cells were lysed (ACK, Lonza) and an enrichment with biotinylated anti-B220 (BD Biosciences), anti-CD3 (BD Biosciences), following of anti-biotin Ab magnet-bead coupled (Miltenyi) and magnetic LS-columns (Miltenyi) was performed to remove spleen lymphocytes and increase the sorting efficacy. Cells were stained with specific markers of populations of interest (Ly6G-BD BioSciences, Ly6C-BioLegend, CD11b-BD BioSciences) and neutrophils (Ly6G^hi^) and inflammatory monocytes (Ly6C^hi^) were sorted using the BD Biosciences FACSAria device. Sorted cells (>97-98% pure) from naive, infected/non-treated and infected/treated mice showed a viability > 90%. They were cultured in U bottom 96-well plates at a density of 4 × 10^6^ cells/ml (1 × 10^6^ cells/250 µl/well) in 10% FBS-containing RPMI medium. After 24 h of cell culture, cell-free supernatants were collected and stored at −20°C, to allow cytokines and chemokines protein release quantification.

### Flow cytometry

Organs of interest were collected to realize immunophenotyping of immune cells. Spleen cell suspensions were obtained by mechanical dissociation in PBS. BM cell suspensions were obtained by dissection and PBS-2%-FBS flushing of tibias and femurs. Cells were stained at 4°C using fluorochrome-conjugated antibodies against: CD3-FITC (e chain) (145-2C11, BD Biosciences), CD8a-BV650 (53-6.7, BD Biosciences), CD11b-AAF (M1/70, eBioscience), CD11c-V450 (HL3, BD Biosciences), CD45.2-V500 (104, BD Biosciences), CD62L-FITC (MEL-14, BD Biosciences), CD86-Pe-Cy7 (GL1, BD Biosciences), Ly6G-PerCPCy5.5 (1A8, BD Biosciences), Ly6C-BV605 (AL-21, BioLegend) and MHCII-FITC (2G9, BD Biosciences). FrCasE-infected cells were assayed using an anti-Gag mAb (H34) [28] labelled with Alexa Fluor 647 (Thermo Scientific). FcγRIV expression was determined using 9E9 antibody (kindly provided by Dr. Pierre Bruhns, Institut Pasteur), produced by BioXcell, and then labelled with Alexa Fluor 647 (Thermo Scientific). Forward scatter area and forward scatter time-of-flight, as well as side scatter, were used to remove doublets from flow cytometry analyses. Cells were analyzed on FACS LSR Fortessa (BD Bioscience), and the data were analyzed using the FlowJo software.

### Neutrophils and monocytes isolated from BM used in in vitro experiments

Neutrophils and monocytes were purified from 8 to 11-week-old naive mice BM. After dissection of lower limbs, BM cell suspensions were collected by PBS-2% FBS EDTA (2 mM) flushing (25G needle) of tibias and femurs. BM cell suspensions were filtered with a 0,40 µm nylon strainer. Two magnetic-based cell sorting (MACS) isolation kits were used to purified either neutrophils (Neutrophil isolation kit, Miltenyi Biotec) and monocytes (Monocyte isolation kit, Miltenyi Biotec) by negative selection, both with high purity (>97-98%), determined by FACS (LSR Fortessa, BD Bioscience). Briefly, non-target cells were magnetically labelled with a cocktail of biotin-conjugated primary mAbs and then an anti-biotin mAb conjugated to MicroBeads as secondary labelling reagent was added. The non-targeted cells were retained in the magnet whereas target cells, neutrophils or monocytes, run through. Cells were placed in culture in U bottom 96-well plates at a concentration of 10^6^/ml in 10% FBS-containing RPMI medium.

### Stimulation of neutrophils and monocytes in vitro

Purified neutrophils and monocytes were seeded at 150 000 cells/ well in 150 µl of RPMI, then incubated for 24 h with LPS (1 µg/ml, Sigma), or FrCasE virus (MOI 5: 5 viral particles /cell), or ICs (virus FrCasE and 1 µg 667 mAb), or 1 µg 667 mAb alone. The MOI and 667 mAb concentration used to form ICs were previously identified by dose–response experiments involving different MOI and different 667 mAb quantities. In parallel, the same experiments were performed adding inflammatory cytokines, TNFα 100 UI/ml (Peprotech), IFN γ 100 UI/ml (eBioScience), IFNα11 1000 UI/ml, produced and generously provided by Dr. Gilles Uzé (DIMP, CNRS). After 24 h of stimulation, supernatants were collected and stored at −20°C to quantify chemokines and cytokines protein release secretion. The viability of neutrophils and monocytes 24 h post-stimulation was high (> 85%). No significant differences in cell viability were observed among the different stimulation conditions. Phenotypic activation of both neutrophils and monocytes was measured using surface markers by flow cytometry.

### Chemokines and cytokines protein release quantification

Soluble chemokines and cytokines secretion were quantified from cell-free collected supernatants of *in vitro* cultured neutrophils and monocytes and of sorted splenic neutrophils and inflammatory monocytes (of naive, infected/non-treated and infected/treated mice 8 and 14 days p.i.), using bead-based immunoassays (LegendPLex, BioLegend) and analyzed on the BD Bioscience-LSR Fortessa device. The protein release quantification was established by the appropriate software (LEGENDplex^TM^ data analysis).

### FcγRIV-blocking mAb (9E9)

Specific FcγRIV*-*blocking mAb (9E9), from Armenian hamster was kindly provided by Dr. Pierre Bruhns (Institut Pasteur, Paris) and was produced by BioXCell. The isotype control antibody (IsoC) (BioXCell) is an IgG1 also from Armenian hamster, directed against GST (Glutathione-S-Transferase). Antibodies were deglycosylated to avoid interaction of its Fc Fragment with other FcγR expressed on FcγRIV-expressing cells [29]. FcγRIV*-*blocking mAb (9E9) or isotype control (IsoC) was administered at the dose of 5 µg/g, every 3 days starting one day before the infection at 8 days post birth, until 21 days p.i. i.e. the time necessary to eliminate the therapeutic 667 mAb.

### ELISA of anti-FrCasE antibodies

Plasma anti-FrCasE IgGs were assayed by ELISA as previously described [13,30,31]. More precisely mice were bled at the retro-orbital sinus to assay anti-FrCasE serum immunoglobulin concentrations. After clotting at room temperature for 15 min, blood samples were centrifuged at 8000× *g* for 10 min in order to isolate serum. Samples were diluted in PBS containing 0.1% Tween 20 and 1% bovine serum albumin. Peroxidase-conjugated anti-mouse IgG1 or anti-mouse IgG2a-specific antisera (Serotec) were used as secondary antibodies. The 667 (IgG2a isotype) and 678 (IgG1 isotype) mAbs [27] were used as control.

### Statistics

Statistical analyses were performed using GraphPad Prism 5 (GraphPad Software). Data were expressed as mean ± SEM, and statistical significance was established using a parametric 1-way ANOVA test with Bonferroni’s multiple comparisons post-tests or non-parametric Kruskal–Wallis test with Dunn’s multiple comparisons post-test for multiple comparisons or paired Student’s *t* tests when two groups were compared. *P* values lower than 0.05 were considered as statistically significant.

## Results

### Neutrophils activated by viral determinants, free or in the form of ICs, secrete high levels of chemokines able to recruit monocytes and neutrophils

**Figure 1.**
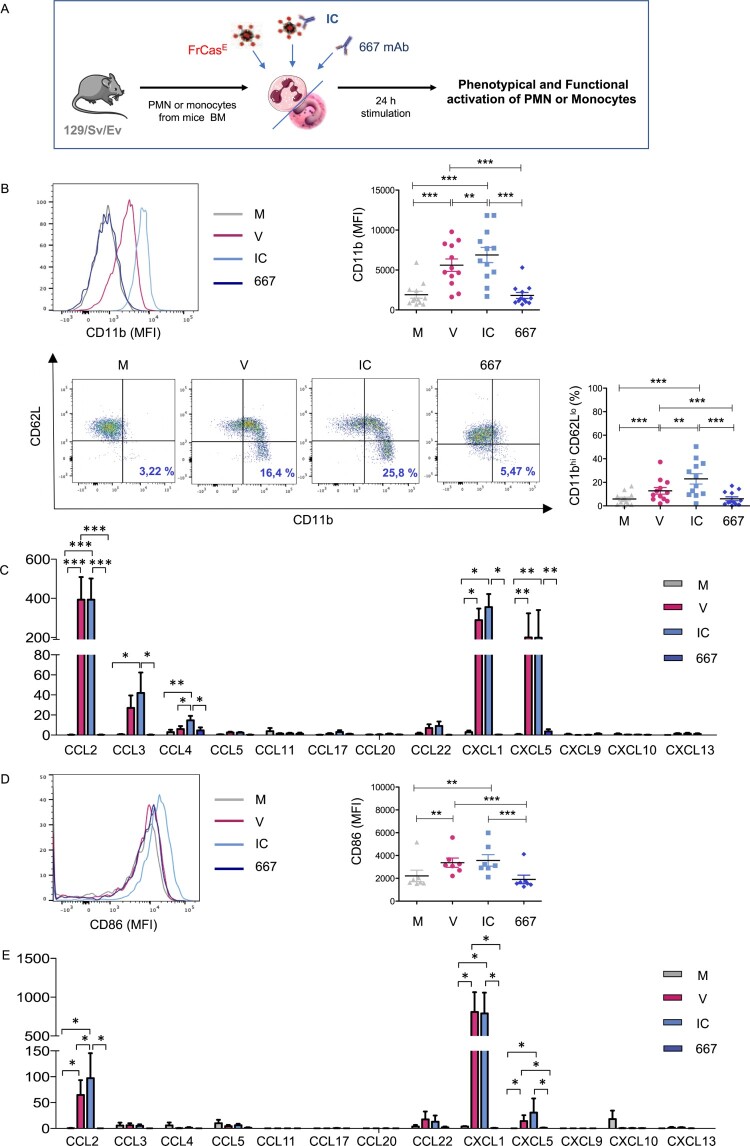
Phenotypic and functional activation of neutrophils and monocytes stimulated with FrCasE virions and ICs. (A). Experimental scheme. V, free virions; IC, viral ICs; M, culture medium. Free 667 mAb was used as control, 667. (B) *Phenotypic activation of neutrophils stimulated by FrCasE virions or viral ICs made with the 667 mAb.* Activation was assessed by monitoring CD11b expression and frequency of CD11b^hi^ CD62L^lo^ neutrophils. The data represent 12 independent experiments. (C) *Functional activation of neutrophils stimulated by FrCasE virions (V) or viral ICs made with the 667 mAb (IC) or free 667 mAb (667).* Chemokines release was assessed in supernatants of neutrophils isolated from BM of naive mice (>97-98% purity) and stimulated for 24 h by FrCasE virions (red), viral ICs (blue), free 667 mAb (dark blue) or left unstimulated (grey). The data represent 5 independent experiments. (D) *Phenotypic activation of monocytes stimulated by FrCasE virions or viral ICs made with the 667 mAb.* Activation was assessed by monitoring CD86 expression. The data represent 7 independent experiments. (E) *Functional activation of monocytes stimulated by FrCasE virions (V) or viral ICs made with the 667 mAb (IC). C*hemokines release was assessed in supernatants of monocytes isolated from BM of naive mice (>97-98% purity) and stimulated for 24 h by FrCasE virions (red), viral ICs (blue), free 667 mAb (dark blue) or left unstimulated (grey). The data represent 5 independent experiments. Data are expressed as means +/- SEM. Statistical significance was established using a parametric 1-way ANOVA test with Bonferroni’s multiple comparisons post-tests (*p < 0.05; **p < 0.01; ***p < 0.001).

We have previously shown a key role of neutrophils in the induction of long-term protective antiviral immunity upon mAb therapy of infected mice [18]. To better characterize the phenotypical and functional activation of neutrophils by viral determinants, free or in the form of ICs, we isolated bone marrow (BM) neutrophils from naive mice and stimulated them for 24 h *in vitro* with free FrCasE virions or opsonized with the 667 mAb (ICs) (Figure 1A). Free 667 mAb was used as control and showed no effect on the activation of neutrophils (Figure 1B–C). Both, FrCasE virions and ICs induced a strong activation of neutrophils as shown by a higher expression of the CD11b molecule as well as an increased frequency of CD11b^hi^ CD62L^lo^ neutrophils (Figure 1B). However, IC-mediated phenotypic activation was significantly higher.

We next assessed the functional activation of virus- and IC-stimulated neutrophils by measuring their capacity to secrete chemokines and cytokines. Both stimuli led to high secretion levels of several chemokines such as CCL2, CXCL1, CXCL5 and to a lesser extent CCL3 and CCL4 (Figure 1C), the secretion of the latter chemokine being significantly enhanced in IC-stimulated neutrophils. However, both the virus and ICs poorly induced the secretion of the 12 cytokines analyzed (Supplemental Figure 1A). This contrasted with the high secretion of IL-6 and TNFα pro-inflammatory cytokines observed upon lipopolysaccharide (LPS) stimulation despite similar CD11b upregulation induced by LPS and viral determinants (Supplemental Figure 1B and C). In addition, LPS-stimulated neutrophils only secreted CCL3 and CCL4 chemokines, with no secretion of CCL2, CXCL1, CXCL5 chemokines (Supplemental Figure 1D). These data show that bacterial-related pathogen-associated molecular patterns (PAMPs) induce a functional activation of neutrophils different from that of viral stimuli. Interestingly, the chemokines which were more strongly secreted by neutrophils upon viral stimuli (but not by LPS) have been shown to be involved in the recruitment of neutrophils themselves (CXCL1, CXCL5) and inflammatory monocytes (CCL2).

### Inflammatory monocytes activated by viral determinants, free or in the form of ICs, secrete high levels of chemokines able to recruit neutrophils and monocytes

We next assessed the activation of Ly6C^hi^ monocytes isolated from naive mice BM and stimulated for 24 h with virus or ICs (Figure 1A). Both stimuli significantly activated monocytes (as depicted by an increased CD86 expression) (Figure 1D) and induced the secretion of CXCL5, CXCL1 and CCL2 chemokines, the secretion of the latter chemokine being significantly enhanced in IC-stimulated monocytes (Figure 1E). As compared to neutrophils, higher amounts of the neutrophil-recruiting chemokine CXCL1 were detected as well as lower amounts of CXCL5 and CCL2. Virus- and ICs induced a weak secretion of most of the 12 cytokines analyzed (Supplemental Figure 2A). This contrasted with high level secretion of IL-6, TNFα and IFNγ observed upon LPS activation (Supplemental Figure 2B and C). LPS stimulation also induced a wider and different panel of chemokine release (Supplemental Figure 2D), notably with the secretion of high amounts of CCL3, CCL4, CCL5, and to a lesser extent CXCL1 and CXCL10. As observed in neutrophils, these data show that viral stimuli induce a functional activation of monocytes different from that of bacterial-related PAMPs.

### Inflammatory conditions potentiate the activation of neutrophils and monocytes by viral ICs

As the inflammatory microenvironment resulting from the viral infection and mAb therapy might affect the antiviral immune response, we next assessed the phenotypic and functional activation of neutrophils and monocytes by viruses and ICs under an inflammatory environment (i.e. in the presence of proinflammatory/immunomodulatory cytokines, such as TNFα, IFN-I and IFNγ). Free 667 mAb in combination with each cytokine was used as control and showed no effect on the secretion profile of either cell type (not shown), similar to free 667 mAb alone (Figure 1).

**Figure 2.**
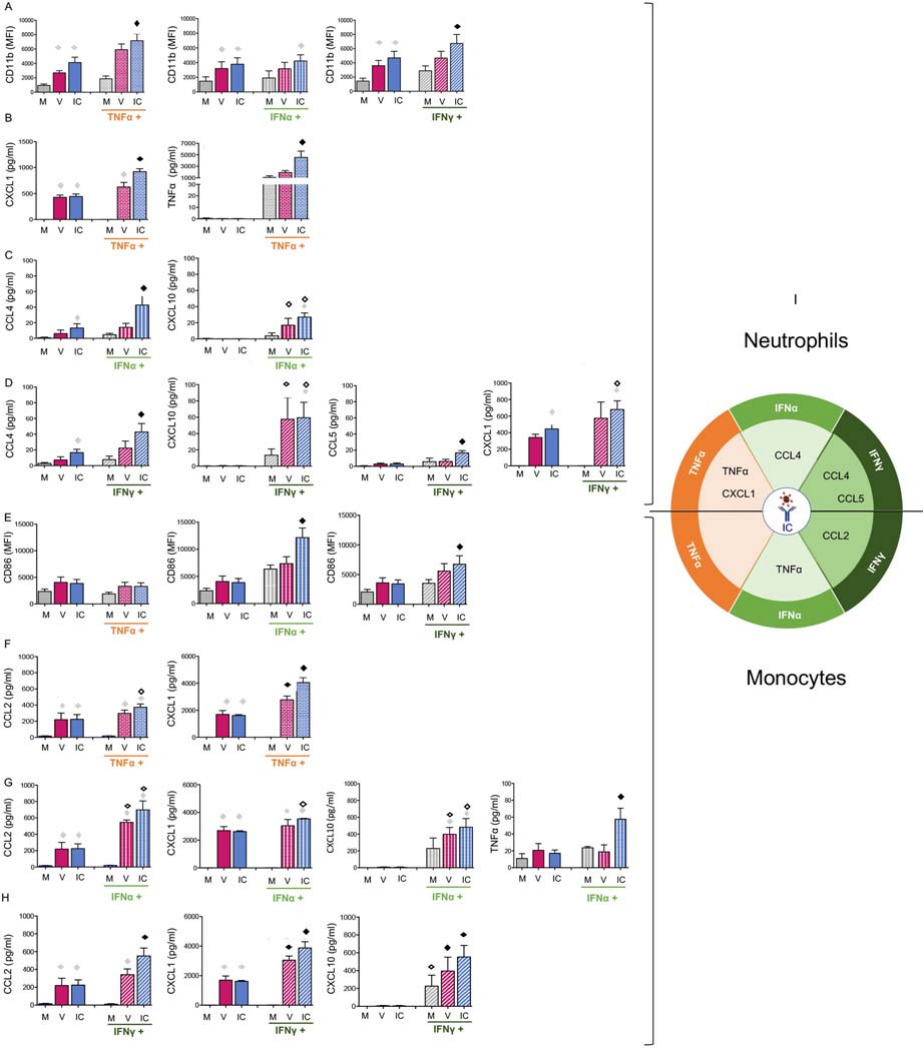
Cytokine stimulation potentialize the functional activation of neutrophils and monocytes by ICs. BM-derived neutrophils and monocytes were isolated from naive mice and activated as in Figure 1 in the presence, or in the absence, of TNFα, IFN-I or IFNγ. V, free virions; IC, viral ICs; M, culture medium. (A-D). *Functional properties of neutrophils activated by TNFα, IFN-I or IFNγ.* Phenotypic activation was assessed by monitoring CD11b (A). Modulation of the functional activation of neutrophils by TNFα (B), IFN-I (C) or IFNγ (D). Chemokines and cytokines release were assessed in supernatants of activated neutrophils (B-D). The data represent 6 independent experiments for A, 4 independent experiments for B-D. (E-H) *Functional properties of monocytes activated by TNFα, IFN-I or IFNγ.* Phenotypic activation was assessed by monitoring CD86 expression (E). Modulation of the functional activation of monocytes by TNFα (F), IFN-I (G) or IFNγ (H). Chemokines and cytokines release were assessed in supernatants of activated monocytes (F-H). The data represent 3 independent experiments for E and 3 independent experiments for F-H. Data are expressed as means +/- SEM. Diamonds indicate significant differences to all the other stimulation conditions (black diamond), or to the corresponding stimuli in the absence of cytokine stimulation (open diamond), to corresponding medium without virus or IC stimuli (grey diamond) as determined by Kruskal-Wallis test with Dunn’s multiple comparisons post-tests (*p* < 0.05). (I) Diagram summarizing the cytokines and chemokines enhanced by TNFα, IFN-I or IFNγ in IC-activated neutrophils and monocytes but not in virus-activated cells.

TNFα and IFNγ significantly enhanced the phenotypic activation of neutrophils by ICs (but not by free virus) (Figure 2A). We also showed that TNFα, IFNγ and IFN-I enhanced the secretion of different cytokines and chemokines by IC-stimulated neutrophils (Figure 2B–D). Notably, we observed an enhanced secretion of TNFα (by TNFα itself), CXCL1 (by TNFα and IFNγ), CCL4 and CXCL10 (by IFNγ and IFN-I) and CCL5 (by IFNγ). In contrast, inflammatory conditions hardly modified the secretion profile of free virus-activated neutrophils as only the secretion of the CXCL10 was induced in virus stimulated cells, consistent with the IFN-dependent induction of this chemokine.

With regard to monocytes, IFN-I and IFNγ significantly potentiated the phenotypic activation of IC-stimulated monocytes (as depicted by an increased expression of the CD86 molecule) (Figure 2E) but not that of virus-activated cells. TNFα, IFNγ and IFN-I also enhanced the secretion of different cytokines/chemokines by IC-stimulated monocytes, leading to increased secretion of CCL2 and CXCL1 (enhanced by the 3 cytokines), TNFα (enhanced by IFN-I) and CXCL10 (enhanced by both types of IFN) (Figure 2F–H). TNFα, IFNγ and IFN-I stimulation also modulated the cytokine/chemokine secretion profile of virus-activated monocytes (i.e. CCL2, CXCL1), although to a lesser extent than IC-activated cells.

Although TNFα, IFNγ and IFN-I generally increased cell activation across several stimulation conditions, these data show that inflammatory conditions potentiate in a significant manner the phenotypic activation of neutrophils and monocytes activated by ICs but not by free virus (Figure 2A and E). Similarly, the secretion of several cytokines and chemokines is significantly enhanced by TNFα, IFN-I or IFNγ in IC-activated neutrophils and monocytes but not in virus-activated cells (Figure 2B–D, Figure 2F–H and Figure 2I). It is worthy of note that the cytokine/chemokine secretion enhancement observed in IC-activated cells differs depending on the stimulating cytokine and the responding cell type (i.e. increased secretion of TNFα, CCL4, CCL5 and CXCL1 by IC-activated neutrophils and CCL2 and TNFα by IC-activated monocytes) (Figure 2I). Overall, our data show that inflammatory conditions have a stronger effect in the enhancement of the secretion profile of IC-activated cells (both in terms of quality and quantity of cytokines/chemokines secreted) as compared to virus-activated cells (notably in neutrophils).

### Inflammatory conditions upregulate the expression of FcγRIV on in vitro activated neutrophils and monocytes

**Figure 3.**
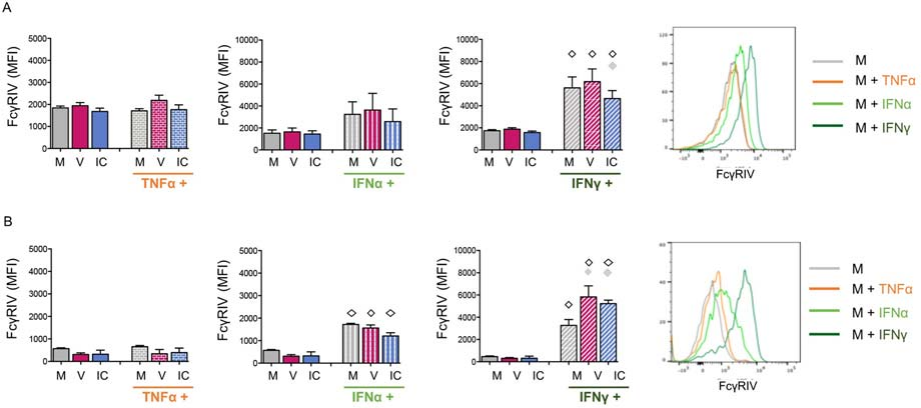
FcγRIV is upregulated by IFN stimulation on both neutrophils and monocytes cell surface. Neutrophils and monocytes were isolated from naive mice and stimulated as in Figure 2. FcγRIV expression was evaluated on neutrophils (A) and monocytes (B). V, free virions; IC, viral ICs; M, culture medium. The data represent 6 independent experiments for neutrophils (A) and 3 independent experiments for monocytes (B). Data are expressed as means +/- SEM. Diamonds indicate significant differences to the corresponding stimuli in the absence of cytokine stimulation (open diamond) or to corresponding medium without virus or IC stimuli (grey diamond) as determined by Kruskal-Wallis test with Dunn’s multiple comparisons post-tests (*p* < 0.05).

FcγRs expression might differ between steady-state *versus* inflammatory/pathological conditions [32], however little is known about modulation of FcγRs expression in the context of viral infections and mAb-therapy. We thus assessed whether the activation of neutrophils and monocytes by virus and ICs affected the expression of the activating receptor FcγRIV, both in the absence and in the presence of inflammatory cytokines. The modulation of this FcγR is all the more relevant to be studied in this experimental model as (i) it is a high affinity receptor for IgG2a, (which is the isotype of the 667 mAb) and (ii) it is highly expressed on neutrophils, the latter having a key immunomodulatory role in mAb-mediated protection of retrovirus-infected mice [18]. We found that IFNγ and IFN-I stimulation (but not TNF-α) led to the upregulation of FcγRIV expression on neutrophils (IFNγ) (Figure 3A) and monocytes (IFNγ and IFN-I) (Figure 3B). In contrast, in the absence of inflammatory cytokines, neither the virus nor the ICs significantly modulated the FcγRIV expression on neutrophils (Figure 3A) and monocytes (Figure 3B). However, activation of monocytes by IFNγ combined with virus or ICs significantly upregulated the expression of FcγRIV as compared to IFNγ alone. These results show the specific effect of the different inflammatory cytokines (TNF-α, IFN-I, IFN-γ) on the modulation of FcγRIV expression.

### Viral infection and mAb therapy upregulate FcγRIV expression on neutrophils and on inflammatory monocytes

**Figure 4.**
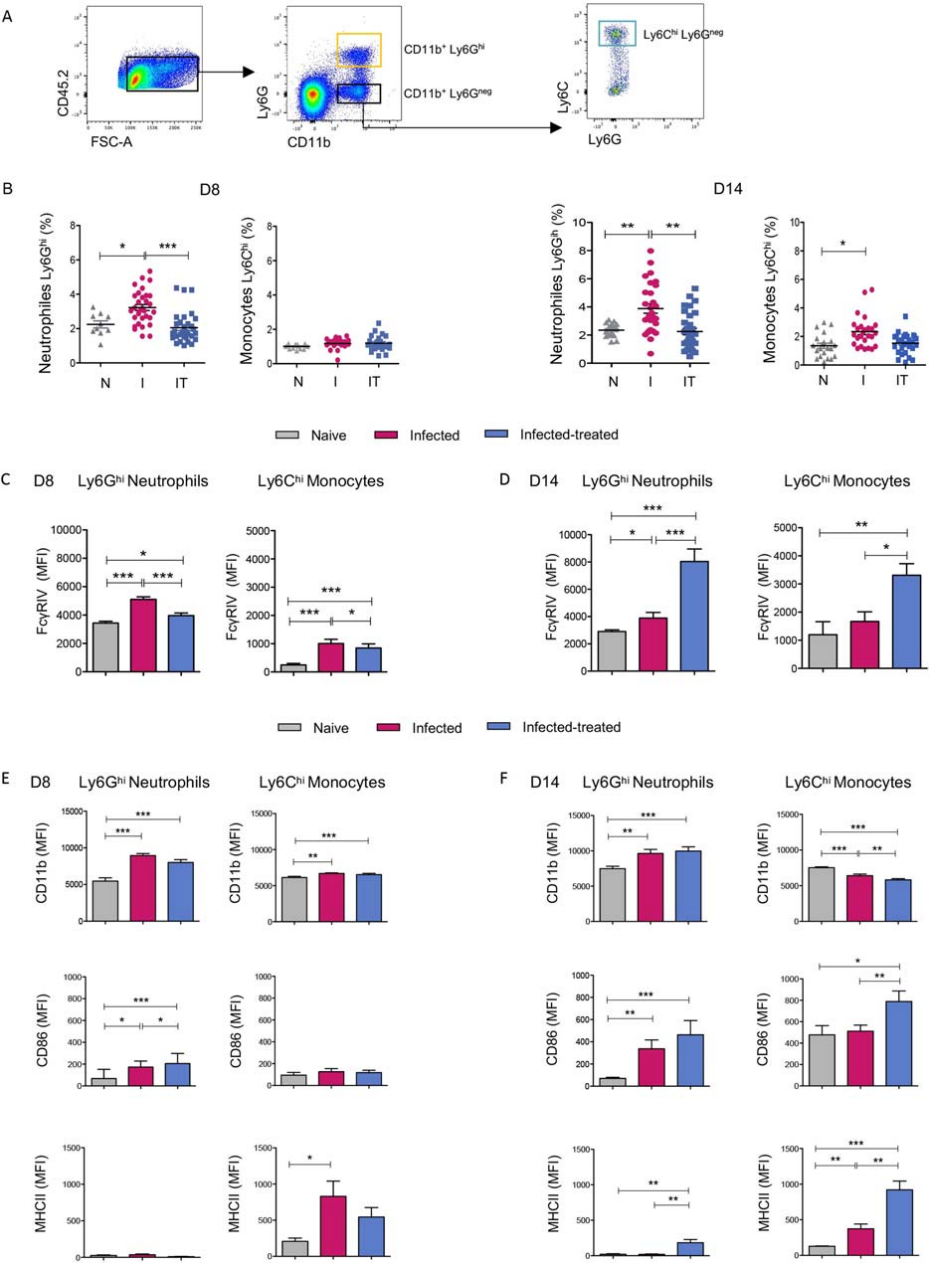
FcγRIV is upregulated *in vivo* on splenic neutrophils and on inflammatory monocytes. Splenocytes from naive (grey), infected/non-treated (I; red) and infected/treated (IT; blue) mice were analyzed on days 8 (D8) and 14 (D14) p.i. for FcγRIV expression. (A). Gating strategy used to define neutrophil and monocyte populations. (B). Frequencies of *CD11b^+^Ly6G^hi^* neutrophils and *Ly6C^hi^* monocytes. (C). FcγRIV expression on *CD11b^+^Ly6G^hi^* neutrophils and *Ly6C^hi^* monocytes. (D). Cell surface markers expression (CD11b, CD86, MHCII) on *CD11b^+^Ly6G^hi^* neutrophils and *CD11b^+^Ly6C^hi^* monocytes surface. The data represent 5 independent experiments at D8 p.i. and 6 independent experiments at D14 p.i with at least 6–8 mice per group (I and IT) and 3–5 mice per group (naive mice). Data are expressed as means +/- SEM. Statistical significance was established using a parametric 1-way ANOVA test with Bonferroni’s multiple comparisons post-tests (*p < 0.05; **p < 0.01; ***p < 0.001).

We next assessed *in vivo* whether the inflammatory environment resulting from FrCasE viral infection and 667 mAb therapy modulated the expression of FcγRIV on neutrophils and inflammatory monocytes. To this end, mice were infected and treated, or not, with the therapeutic mAb (infected/treated and infected/non-treated, respectively). Then, the expression of FcγRIV on neutrophils and inflammatory monocytes from the spleen (one of the main sites of viral replication) was evaluated at different time points post-infection (p.i.): at day 8 p.i. (when viral replication reach maximal levels in infected/non-treated mice) [13] (Supplemental Figure 3A) and day 14 p.i. (i.e. corresponding to the peak of primary cytotoxic T-cell responses) [13,18]. Age-matched naive mice were used as controls. The cell populations of interest were defined by flow cytometry (Figure 4A) based on the expression of CD11b, Ly6G and Ly6C to gate neutrophils (CD11b^+^, Ly6G^hi^) and inflammatory monocytes (CD11b^+^Ly6G^-^Ly6C^hi^). Neutrophil abundance in infected/treated mice was comparable to that observed in naive mice, whereas it was significantly higher in infected/non-treated animals at days 8 and 14 p.i. (Figure 4B). Increased neutrophil frequency was associated with a higher percentage of spleen infected cells (Supplemental Figure 3A), as we previously described [18]. However, inflammatory monocytes abundance was only significantly increased in infected/non-treated mice at day 14 p.i. (Figure 4B). Higher frequencies of NK cells, CD4^+^ T cells, CD8^+^ T cells and CD11c^+^ DC were also detected in infected/non-treated animals at days 8 and 14 p.i. as well as in infected/treated mice at day 14 p.i. (Supplemental Figure 3B). Interestingly, significantly higher frequencies of IFNγ/TNFα-producing CD4^+^ and CD8^+^ T cells were observed in infected/treated mice at day 14 p.i. (Supplemental Figure 3C).

Regarding FcγRIV expression, in steady-state conditions, it was highly expressed on splenic neutrophils while lower FcγRIV expression was detected in Ly6C^hi^ monocytes at the different time points assessed (Figure 4C and D). At day 8 p.i. FcγRIV expression was significantly upregulated on neutrophils and monocytes in infected/non-treated mice. In contrast, at day 14 p.i. infected/treated mice showed a stronger upregulation of FcγRIV on both cell-types as compared to infected/non-treated and naive mice. These results suggest a distinct inflammatory environment in infected/non-treated mice *versu*s infected/treated mice that evolves over time and differentially modulates the expression of the activating FcγRIV on neutrophils and monocytes.

### FcγRIV upregulation in infected/treated mice is associated with enhanced expression of MHC-II and co-stimulatory molecules on neutrophils and monocytes

We next addressed the activation of splenic neutrophils and inflammatory monocytes in infected mice with or without immunotherapy at days 8 and 14 p.i. by monitoring cell surface activation markers. Similar to FcγRIV expression, we showed that the activation state of the cells evolved over time. At day 8 p.i. (Figure 4E), CD11b was upregulated on neutrophils and inflammatory monocytes upon viral infection either in the absence or in the presence of immunotherapy. However, infected/treated mice showed a significantly higher CD86 upregulation on neutrophils than infected/non-treated mice. At day 14 p.i. (Figure 4F), CD11b and CD86 expression was increased on splenic neutrophils isolated from both infected/treated- and infected/non-treated mice, with no significant differences between these two groups of mice. In contrast, MHC-II upregulation was only induced in infected/treated mice, suggesting a mAb-mediated upregulation of this molecule. Similarly, we observed a significantly higher upregulation of CD86 co-stimulatory molecule and MHC-II on inflammatory monocytes from infected/treated mice as compared to infected/non-treated mice (Figure 4F).

These data show that mAb treatment leads to the upregulation of MHC-II on neutrophils and monocytes at 14 days p.i. as well as the upregulation of the CD86 costimulatory molecule on monocytes (Figure 4E and F). This is associated with the FcγRIV upregulation observed on neutrophils and Ly6C^hi^ monocytes from infected/treated mice at this time point (Figure 4C and D).

### Neutrophils and inflammatory monocytes are differentially and sequentially activated upon antiviral mAb treatment

**Figure 5.**
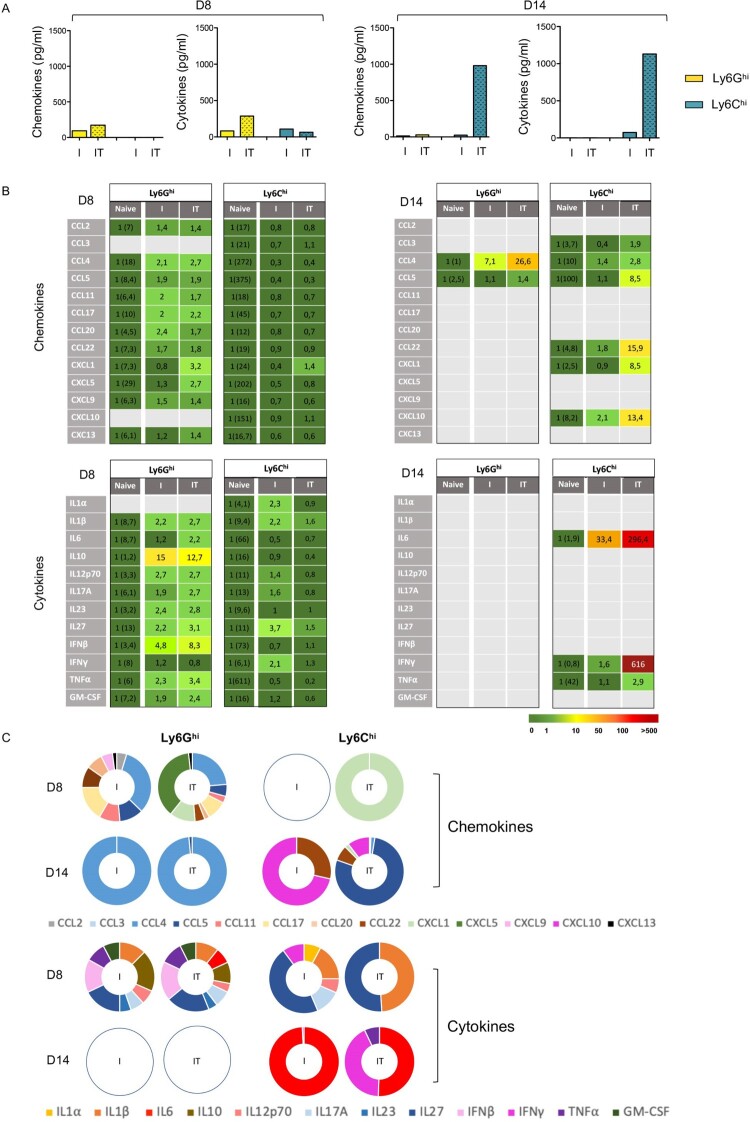
Cytokine and chemokine secretion profile of splenic neutrophils and inflammatory monocytes. Splenic neutrophils and inflammatory monocytes from naive, infected/non-treated (I) and infected/treated mice (IT) were isolated at days 8 p.i. (D8) and 14 p.i. (D14) for assaying their chemokine and cytokine secretion profile in supernatants of sorted cells cultured at a density of 4 × 10^6^ cells/ml (1 × 10^6^ cells/well) for 24 h. Data represent 3 independent experiments on D8 p.i. and 4 independent experiments on D14 p.i. with at least 6–8 mice per group. (A). Total quantity of chemokines and cytokines secreted by neutrophils and inflammatory monocytes from infected/non-treated (I) and infected/treated mice (IT). (B). Chemokine and cytokine secretion of neutrophils and inflammatory monocytes isolated from naive, infected/non-treated (I) and infected/treated mice (IT). The colour code shows fold-increase compared to secretion detected in cells isolated from naive mice (the raw chemokine and cytokine release values are given in brackets, expressed in pg/ml). (C). Chemokine and cytokine secretion of neutrophils and inflammatory monocytes isolated from infected/non-treated (I) and infected/treated mice (IT) expressed as percentage of chemokine and cytokine showing a fold-increase ≥1.4 as compared to cells isolated from naive mice.

To further characterize the activation state of neutrophils and Ly6C^hi^ monocytes upon viral infection and mAb-therapy, we addressed the cytokine and chemokine secretion profile of splenic neutrophils and monocytes sorted from infected mice treated, or not, with the 667 mAb, at days 8 and 14 p.i. Cells sorted from age-matched naive mice were used as controls. We found that the secretion profile of neutrophils and monocytes was distinct but it was globally enhanced in both cell-types in infected/treated mice as compared to infected/non-treated mice (Figure 5A and B), in particular at day 8 p.i. in neutrophils and at 14 p.i. in monocytes.

At day 8 p.i., neutrophils from both infected/non-treated and infected/treated mice showed a broad chemokine and cytokine secretion profile as deduced from an increased secretion of most of the 13 chemokines and 12 cytokines assessed, although at weak levels (Figure 5B and C). Notably, neutrophils from infected/treated mice showed a higher increase in IFN-I (IFNβ) secretion (8,3-fold increase) than neutrophils from infected/non-treated mice (4,8-fold increase). In contrast, infection and mAb-treatment hardly affected the chemokine/cytokine secretion of Ly6C^hi^ monocytes as they only showed a weak increase (1.4-fold increase) in CXCL1 secretion in infected/treated mice as well as a slight increase in cytokines secretion (both in terms of diversity and fold increase) mainly in infected/non-treated mice (Figure 5B).

Interestingly, at day 14 p.i., the functional activation of both cell-types completely differed from that observed at day 8 p.i. (Figure 5B and 5C). We found a more restricted but stronger induction of chemokines/cytokines secretion, mostly in cells isolated from infected/treated mice. In contrast to the broad cytokines and chemokines secretion profile observed at day 8 p.i., neutrophils only secreted CCL4 and CCL5 chemokines and showed no cytokine secretion at day 14 p.i. It is worth noting that CCL4 secretion was strongly enhanced in infected/treated mice consistent with the enhanced secretion of this chemokine observed upon neutrophils IC-stimulation *in vitro*. In addition, Ly6C^hi^ monocytes showed a higher secretion of 6 chemokines (CCL3, CCL4, CCL5, CCL22, CXCL1 and CXCL10) and 3 Th1-polarizing cytokines (IL-6, TNFα, IFNγ) in infected/treated mice as compared to infected/non-treated mice, with notably a strong induction of IL-6 and IFNγ. These data suggest a role for the therapeutic mAb in the functional activation of these FcγRIV-expressing cells.

### The enhanced secretion of chemokines and Th1-polarizing cytokines by neutrophils and Ly6C^hi^ monocytes in infected/treated mice occurs in a FcγRIV-dependent manner

We next evaluated the role of FcγRIV in the modulation of the functional properties of neutrophils and inflammatory monocytes in infected/treated mice. To this aim, we used a highly specific FcγRIV-blocking mAb (the 9E9 mAb, [33]) in a deglycosylated form to avoid its binding to other FcγRs through the Fc fragment [29] (Supplemental Figure 4). An irrelevant mAb of the same isotype (IsoC) was used as control. The 9E9 or IsoC were administered every 3 days starting one day before the infection until the functional activation of neutrophils and monocytes was assessed (at day 14 p.i.) (Supplemental Figure 4A). FcγRIV-blocking was specific and efficient (Supplemental Figure 4B-4C) and did not affect the frequencies of splenic neutrophils and monocytes (Supplemental Figure 4D).

**Figure 6.**
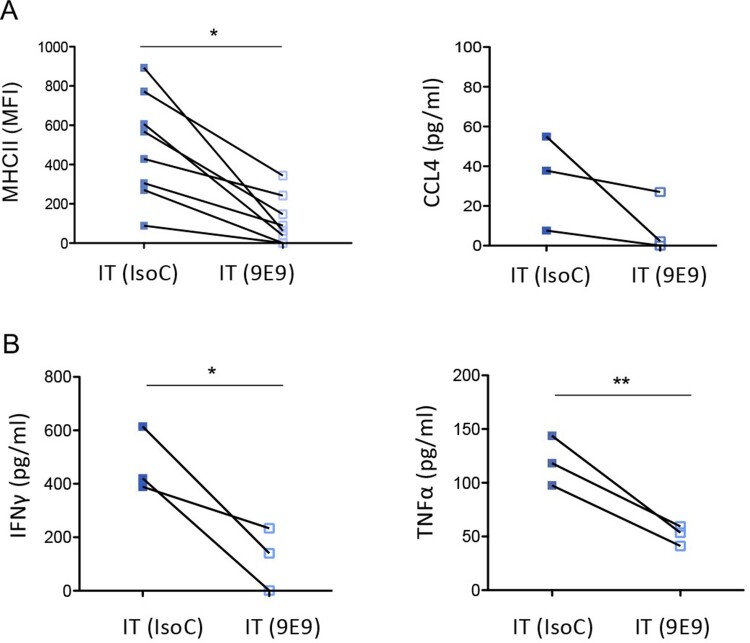
Effect of FcγRIV-blocking on the functional activation of splenic neutrophils and Ly6C^hi^ inflammatory monocytes. Splenic Ly6G^hi^ neutrophils and Ly6C^hi^ monocytes from infected/treated mice (IT), treated with FcγRIV-blocking (9E9) or not (IsoC), were isolated at day 14 p.i. for assaying their functional activation. (A-B). Functional activation of neutrophils (A) and monocytes (B). MHC-II expression on neutrophils was assessed by flow cytometry and the secretion of chemokines and inflammatory cytokines was evaluated in supernatants of sorted neutrophils (A) and monocytes (B) cultured at a density of 4 × 10^6^ cells/ml (1 × 10^6^ cells/well) for 24 h. Data are expressed as means +/- SEM. Statistical significance was established using a paired Student’s *t* test (*p < 0.05; **p < 0.01).

The administration of the FcγRIV-blocking mAb in infected/treated mice led to reduced MHC-II expression on neutrophils at day 14 p.i. (Figure 6A). Furthermore, it also led to a decreased secretion of chemokines (CCL4) and Th1-polarizing cytokines (TNFα, IFNγ) by neutrophils and inflammatory monocytes, respectively (Figure 6A and B). These observations show that FcγRIV triggering is involved in the enhancement of the functional activation of both cell-types.

### FcγRIV-blocking in infected/treated mice leads to reduced immune protection

**Figure 7.**
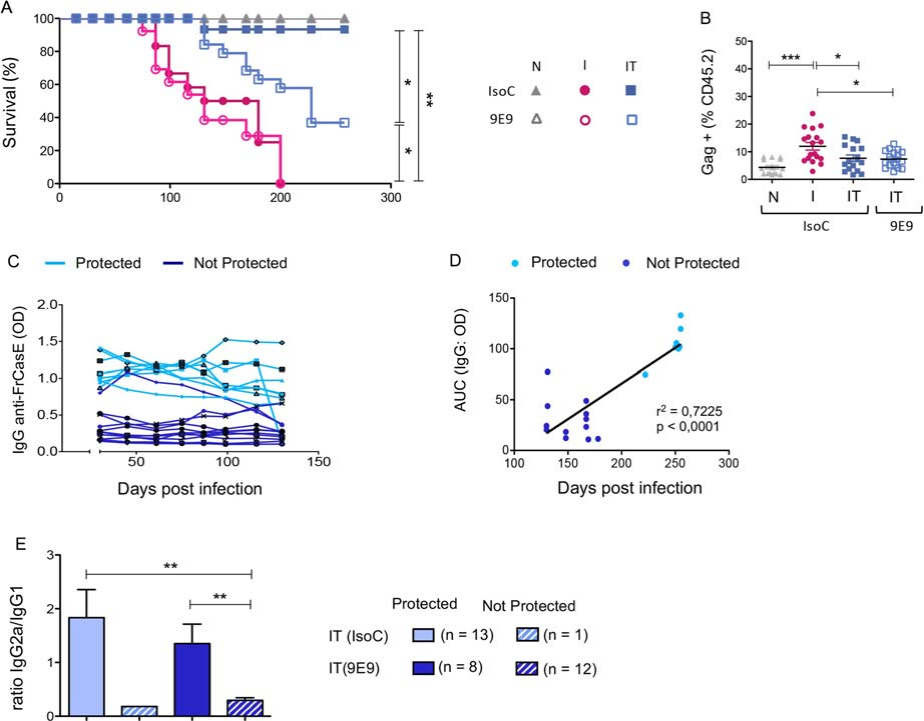
Effect of FcγRIV-blocking on the immune protection of infected/treated mice*.* (A). *Effect of FcγRIV-blocking on mice survival:* naive (N; grey triangle), infected/non-treated (I; red circle) and infected-treated (IT; blue square) mice, with either FcγRIV*-*blocking mAb (9E9; empty shapes) or isotype control (IsoC; full shapes). Results represent 2 independents experiments with 6–11 animals per group of infected/non-treated (I) and infected treated (IT) mice, and 4–5 per group of naive mice. (B). *Effect of FcγRIV-blocking on the viral propagation*. Splenocytes from naïve (N), infected/non-treated (I) and infected/treated (IT) mice, treated or not with FcγRIV-blocking mAb antibody, were analyzed by flow cytometry on day 14 p.i. for retroviral positivity of splenocytes (percentage of Gag^+^ cells) gated in the CD45.2^+^ population. Data represent 4 independent experiments, with at least 4–5 mice (I and IT) per group and 2–3 mice for control group. Statistical significance was established using a parametric 1-way ANOVA test with Bonferroni’s multiple comparisons post-tests (C). *FrCasE specific humoral response in infected/treated mice upon FcγRIV-blocking.* Anti-FrCasE IgG production was quantified over time, by ELISA, for each group of infected/nontreated, and infected/treated mice. (D) *Correlation between serum anti-FrCasE IgG levels and survival times in infected/treated mice upon FcγRIV-blocking*. Serum anti-FrCasE IgG levels were evaluated as area under curve (AUC). Data were analyzed using the Pearson correlation test. (E) *High IgG2a /IgG1 ratio is associated with disease protection in infected/treated mice*. Serum anti-FrCasE IgG1 and IgG2a levels at day 72 p.i. were quantified by specific ELISA. All infected treated/mice with FcγRIV-blocking (9E9) (n = 20), or not (IsoC) (n = 14) were evaluated for such a correlation for virus-specific IgG2a /IgG1 ratio*.* The data represent 2 independent experiments. Data are expressed as mean ± SEM. Statistical significance was established using a parametric 1-way ANOVA test with Bonferroni’s multiple comparisons post-tests. (*p < 0.05; **p < 0.01; ***p < 0.001).

We previously showed that FrCasE-infected mice develop non-protective immune responses and eventually die of leukemia. In contrast, 667 mAb treatment of FrCasE-infected mice induces long-term protective immunity (i.e. enhanced humoral and cellular antiviral immune responses) through Fc-FcγRs interactions [13,14,18]. The reduced cytokine and chemokine secretion by neutrophils and monocytes in infected/treated mice observed upon FcγRIV blocking at day 14 p.i. suggested a role for this FcγR in mAb-induced protection of infected mice. To address this issue, we assessed the clinical and immunological consequences of FcγRIV***-***blocking in infected/treated mice on the long-term. FcγRIV***-***blocking started 1 d before infection (Supplemental Figure 4A) and was maintained for 21 days, i.e. the time necessary to eliminate the therapeutic 667 mAb. No differences in the survival rate were detected in infected/non-treated mice in the presence or in the absence of FcγRIV-blocking mAb. By contrast, the administration of FcγRIV-blocking mAb reduced the protection provided by 667 mAb to infected mice (Figure 7A). The decreased survival was not associated with lack of viral propagation control as we observed no differences in the frequency of infected cells in the spleen following FcγRIV-blocking (Figure 7B). These data suggest that FcγRIV is not crucial to control the viral spread in infected/treated animals. They are consistent with our previous work showing that NK cells (that do not express FcγRIV) are key in controlling viral propagation by 667 mAb–mediated ADCC [18,34], while FcγRIV-expressing neutrophils have a limited effect on viral propagation in infected/treated mice but are crucial for the generation of long-term protective humoral responses [18]. Based on these observations, we assessed the effect of FcγRIV-blocking on the magnitude and quality of the humoral immune response in infected/treated mice. Interestingly, mice that were not protected by 667 mAb treatment (Figure 7A) showed low levels of anti-FrCasE IgGs (Figure 7C and D). In contrast, the reduced percentage of mice that survived, showed a higher antiviral humoral response than unprotected mice (Figure 7C and D), consistently with the correlation between anti-FrCasE IgG serum levels and long-term protection we previously reported [18,34]. Furthermore, the lack of protection in infected/treated mice upon FcγRIV-blocking was also associated with a low IgG2a/IgG1ratio (< 0,5). By contrast, all protected mice showed a high IgG2a/IgG1 ratio (>1,5), which is associated with a Th1-type immune response (Figure 7E) [13,18,30].

These data show a role for FcγRIV in mAb-mediated protection. Furthermore, the low humoral response and the decreased IgG2a/IgG1 ratio observed in unprotected infected/treated mice in the presence of FcγRIV blocking suggest that this FcγR is involved in the induction of a protective humoral response (predominantly of the IgG2a isotype). These observations are in agreement with the enhanced functional activation of neutrophils and monocytes (i.e. secretion of Th1-type polarizing cytokines, upregulation of MHC-II molecules) observed in infected/treated mice in a FcγRIV-dependent manner.

## Discussion

We have previously shown that neutrophils have a key immunomodulatory role in the induction of protective immunity by antiviral mAbs through the acquisition of B-cell helper functions [18]. Here we provide a new insight into the immunomodulatory role of neutrophils in a context of antiviral mAb-therapy. Our work shows that antibody therapy shapes neutrophils properties, notably it leads to the upregulation of MHC-II expression and the enhanced secretion of multiple cytokines and chemokines. We also show that mAb-treatment strongly enhances the functional activation of inflammatory monocytes leading to the secretion of Th1-type polarizing cytokines and chemokines. These enhanced immunomodulatory functions of neutrophils and monocytes observed in infected/treated mice are associated with the upregulation of FcγRIV upon mAb treatment. Importantly, FcγRIV-blocking in infected/treated mice leads to decreased secretion of cytokines and chemokines by both myeloid cell-types as well as to reduced mAb-mediated protection.

Our data show that neutrophils’ cytokine/chemokine secretion profile differs between viral *versus* bacterial stimulus. This is important to highlight as most of the studies investigating the immunomodulatory role of neutrophils have been conducted in bacterial infection models. Thus, *in vitro* stimulation of neutrophils by viral determinants led to a poor production of cytokines but to a wide and strong release of chemokine, with notably high secretion levels of the monocytes-and neutrophils-recruiting chemokines (CCL2, CXCL1, CXCL5) that was not observed upon LPS stimulation. On the contrary, LPS-stimulated neutrophils produced high amounts of proinflammatory cytokines but a narrower and weaker chemokine release. As for neutrophils, viral determinants also lead to different functional activation of monocytes than LPS. Thus, stimulation of monocytes by viral determinants (but not LPS) led to the secretion of high amounts of the neutrophils-recruiting chemokine CXCL1 and to a lesser extent the monocytes-recruiting chemokine CCL2. These results suggest a self-sustaining mechanism of neutrophils and monocytes recruitment upon viral infection, and raise the hypothesis of an early cooperation between neutrophils and monocytes in the induction of the antiviral immune response. This secretion profile of neutrophils induced by viral determinants is in agreement with an increased CCL2 release observed upon *in vitro* HIV stimulation of neutrophils [35]. Increased levels of CCL2 and CXCL1 have also been reported in neutrophil- and monocytes-infiltrated tissues in different viral infections [36–39]. However, chemokine increase was mostly assessed in total tissue extracts but not directly in neutrophils or monocytes isolated cells, which prevented the identification of the cell origin of chemokines. It is worth noting that a very early (i.e. 24-48 h p.i.) but transient expression of CCL2 and CXCL1 has also been reported upon infection by RSV, CMV and influenza virus [36,37,40]. Such early but transient expression would be in agreement with the very low secretion of these chemokines detected at day 8 p.i. in infected mice treated, or not, with the therapeutic mAb.

Another important issue highlighted in our study is the effect of inflammatory conditions on the modulation of the functional activity of neutrophils and monocytes. Thus, the *in vitro* stimulation with inflammatory/immunomodulatory cytokines potentiated the release of several chemokines and cytokines by IC-activated neutrophils and monocytes (Figure 2) (i.e. CCL2, CCL4, CCL5, CXCL1, TNFα) while having a less pronounced or no effect on virus-activated cells (Figure 2I). This is consistent with the upregulation of FcγRIV also observed in cytokine-stimulated neutrophils and monocytes. Although not formally shown, these results support a role for the inflammatory environment and IC-stimulation in the enhancement of chemokine/cytokine secretion by neutrophils and monocytes observed in infected-treated mice. Our *in vitro* observations also allowed us to dissect the specific effect of IFNγ, IFN-I and TNFα on the enhancement of the functional properties of IC-activated neutrophils and monocytes (both in terms of secretion profile and FcγRIV modulation). Notably, IFNγ and IFN-I stimulation (but not TNF-α), led to the upregulation of FcγRIV on neutrophils and/or monocytes, in agreement with previous reports in other experimental settings [41–43]. This argues in favour of a role for both types of IFN in the upregulation of FcγRIV on neutrophils and monocytes observed *in vivo* upon infection and mAb-treatment. However, FcγRIV expression was more strongly enhanced in infected/non-treated mice at day 8 p.i. and in infected/treated mice at day 14 p.i. suggesting a different and evolving inflammatory environment in both groups of mice. Similarly, the neutrophils and monocytes secretion profiles were also distinct at days 8 and 14 p.i. The different activation state of these cells observed between infected/treated versus infected/non-treated mice (notably at day 14 p.i.) suggests that the control of viral propagation by the therapeutic mAb dramatically changes the inflammatory environment and the subsequent immune outcome. Thus, in addition to blunt viral propagation, Fc-mediated clearance of opsonized virus/infected cells by immune effector cells (i.e. ADCC, CDC, ADCP, …) might generate danger signals able to induce inflammation. This, together with FcγR-triggering might lead to enhanced activation of FcγR-expressing cells. Our *in vivo* results showing FcγRIV upregulation on neutrophils and monocytes are in agreement with the FcγRs modulation induced by the inflammatory conditions resulting from bacterial and IC-mediated autoimmune pathologies [32,44]; and provide new evidence on the regulation of FcγRs expression in the specific inflammatory context of antiviral mAb-based immunotherapies, not reported thus far.

Our work provides new mechanistic insights into the hitherto underestimated role of neutrophils as key cells in the modulation of adaptive antiviral immunity upon mAb treatment. Interestingly, at steady-state conditions, neutrophils express high levels of FcγRIV. Its expression is higher than that observed in inflammatory monocytes. Thus, it is tempting to speculate that upon viral infection and mAb-treatment, the early recruitment of neutrophils, together with their high expression of FcγRIV, result in potent Fc-triggering by ICs in these cells. This might be key to initiate the modulation of immune responses through the production of multiple cytokines and chemokines able to recruit and/or activate multiple innate immune cells such as monocytes, NK cells, DC and neutrophils themselves [22]. This suggests a potential role for neutrophils as early drivers of the induction of vaccinal effects by mAbs. Supporting this hypothesis, at day 8 p.i. neutrophils showed a higher and a wider induction of chemokines and cytokines release than monocytes, while monocytes secreted strong quantities of Th1-polarizing cytokines and chemokines at day 14 p.i. Consistent with this, a differential and sequential functional activation of myeloid cells has been recently reported by Zhang and collaborators in a model of influenza infection [45]. By using single-cell RNA sequencing, they showed two waves of inflammation, with neutrophils being the major contributor to the first wave while macrophages generated a second wave of proinflammatory factors. However, the contribution of the different myeloid cells to the inflammation process was only assessed at the transcription level.

Our work suggests that the upregulation of FcγRIV on neutrophils and inflammatory monocytes induced by the viral infection and mAb-treatment might increase Fc-triggering by ICs leading to improved immune responses, both in terms of magnitude and quality. In agreement with this, the FcγRIV upregulation on neutrophils and monocytes in infected/treated mice observed at 14 days p.i. is associated with (i) higher expression of CD86 costimulatory molecule and MHC-II and (ii) higher secretion of cytokines and chemokines, which might enhance antiviral immune responses. Furthermore, FcγRIV-blocking in infected/mice significantly decreased the functional activation of both myeloid cell-types which was associated with the development of impaired humoral responses and reduced protection against disease, suggesting a contribution of FcγRIV expression on neutrophils and inflammatory monocytes in mAb-mediated immune protection. These observations highlight a key immunomodulatory role of this FcγR in the induction of antiviral immune responses, thus far unreported. Indeed, the involvement of FcγRIV in mAb-mediated protection has mainly been assessed in cancer models [46–48]. In addition, most of the studies only evaluated the effector role of this FcγR. Nevertheless, the contribution of FcγRIV in the induction of protective cellular immune responses upon mAb-treatment has been shown in a mouse model of lymphoma [49]. However, the mechanisms involved in the modulation of the antitumoral response were not evaluated. Similar to our findings, FcγRIV deficiency led to partial protection [49], raising the possibility that other FcγRs might also be involved in mAb-mediated immune protection. This is likely to be the case taking into account the functional redundancy of different FcγRs reported in several experimental settings [47,50,51]. Addressing this issue will require further investigation.

The FcγRIV-dependent high secretion of Th1-polarizing cytokines (TNFα, IFNγ) [52–56] by inflammatory monocytes from infected/treated mice at day 14 p.i, argues in favour of a mAb-mediated Th1 polarization of the antiviral immune response by these cells. Consistent with this, upon FcγRIV-blocking unprotected infected/treated mice showed a low virus-specific IgG2a/IgG1 ratio in contrasts to the high IgG2a/IgG1 ratio (which is associated with a predominant Th1-type immune response) observed in protected mice. These observations are in agreement with our previous work reporting a Th1-biaised immune response in 667-mAb treated, FrCasE infected mice as observed by the development of cytotoxic CD8 T-cell responses [13,18,30] and protective humoral immune responses predominantly of the IgG2a isotype [13,18,26], both occurring in a Fc-dependent manner. Our results are also consistent with the work of Fox and collaborators showing that the therapeutic activity of mAbs against chikungunya virus (CHIK-V) requires Fc-FcγR interaction on monocytes [10]. In agreement with our data, mAb-treatment led to higher levels of proinflammatory chemokines and cytokines in ankles of CHIK-V infected mice. However, chemokines and cytokines were assessed in total ankle samples containing multiples myeloid cell-types (including neutrophils and monocytes) but not in isolated myeloid cells. Thus, neither the effect of mAb-treatment on the secretion profile of monocytes nor the cell origin of chemokines and cytokines were addressed. Finally, it is also worth mentioning the upregulation of MHC-II molecules observed on neutrophils sorted from infected/treated mice. This observation broadens the immunomodulatory role of neutrophils upon mAb-treatment as it suggests that neutrophils might acquire antigen-presenting cell features. In agreement with this, it has been shown that upon *ex vivo* stimulation with IgG immune complex (IC) or viral cognate antigens, neutrophils upregulated expression of MHC-II and costimulatory molecules [57–59]. Consistent with our FcγRIV-blocking results, the MHC-II upregulation in neutrophils observed in these studies required FcγR cross-linking.

Our work shows a distinct secretion profile of neutrophils and Ly6C^hi^ monocytes, in terms of type, amount and kinetics of chemokines/cytokines secretion. This highlights a differentially and sequentially activation of these FcγRIV-expressing cells upon antiviral mAb treatment and suggests their key and complementary action in the induction of protective immune responses by mAb. This is important to keep into consideration as thus far, mostly DC have been considered as key cells involved in the induction of vaccinal effects by mAbs [13,16,17]. Thus, the identification of neutrophils and inflammatory monocytes as players in the induction of protective immunity by mAbs might have therapeutic implications. Importantly, due to their huge therapeutic potential, antiviral mAb are increasingly being considered as antiviral drugs to treat chronic and emerging viral diseases [1,2,6,9,11,60–62], including health emergencies such as Ebola virus [11], Middle East Respiratory Syndrome Coronavirus (MERS-Co) [61] and Severe Acute Respiratory Syndrome Coronavirus 2 (SARS-CoV2) [62] outbreaks. However, a better understanding of the multiple mechanisms of action of antiviral mAbs is still needed to achieve an optimal therapeutic effect. Our findings dissecting the immunomodulatory role of neutrophils and monocytes might thus help to improve mAb-based antiviral therapies by tailoring therapeutic interventions aiming at harnessing the immunomodulatory properties of these cells. To this end, different approaches could be envisaged: (i) the use of cell-type specific, appropriate immunostimulatory host-directed therapies and (ii) the design of antiviral mAbs engineered to enhance their affinity for FcγRs expressed on human neutrophils and monocytes, such as FcγRIIa and FcγRIIIa [17,49,63,64]. Thus, in addition to allow superior antibody-mediated phagocytosis, these Fc-engineered mAbs could also modulate cytokine/chemokine production to ultimately lead to more effective adaptive immune responses.

## Supplementary Material

Supplemental Material

Supplemental Material
