# Supplementary figures and images for: Differential and sequential immunomodulatory role of neutrophils and Ly6Chi inflammatory monocytes during antiviral antibody therapy
Source: Emerg Microbes Infect. 2021 May 21;10(1):964–81. doi: 10.1080/22221751.2021.1913068 (PMC8158214; doi:10.1080/22221751.2021.1913068)

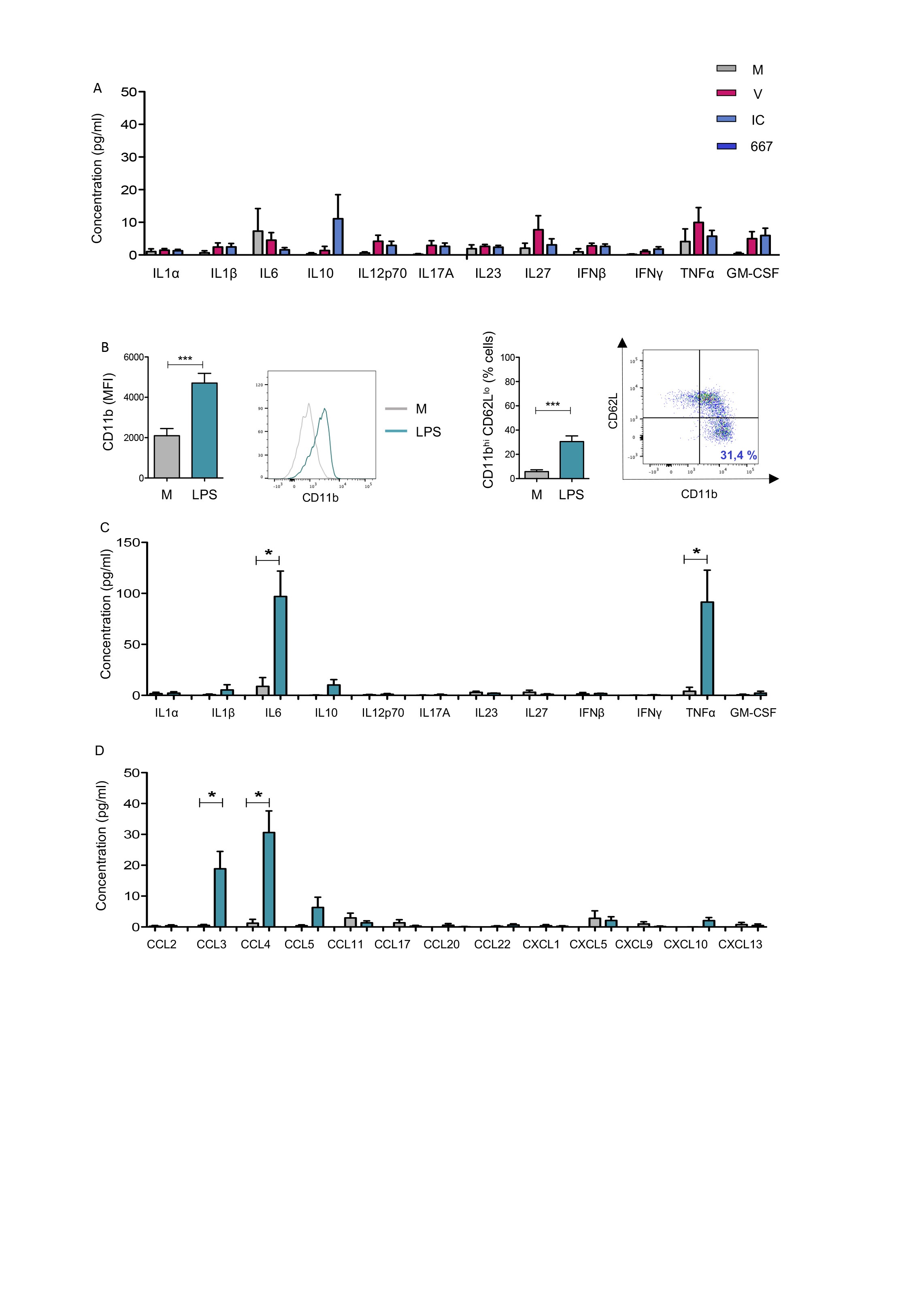

Supplement: Supplemental Material [file TEMI_A_1913068_SM2085.zip › Supplemental Figures/Suppl Fig 1.jpg]

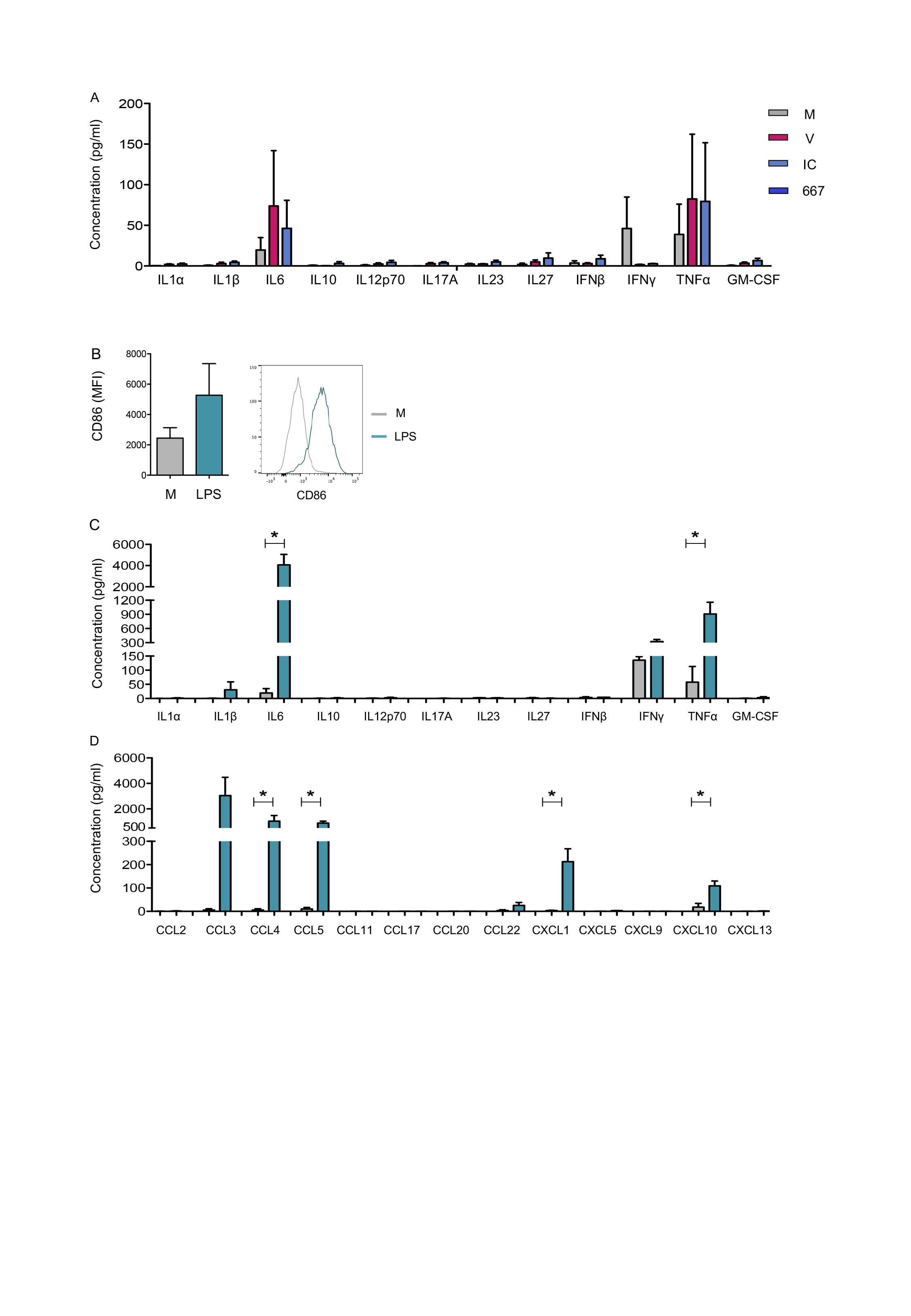

Supplement: Supplemental Material [file TEMI_A_1913068_SM2085.zip › Supplemental Figures/Suppl Fig 2.jpg]

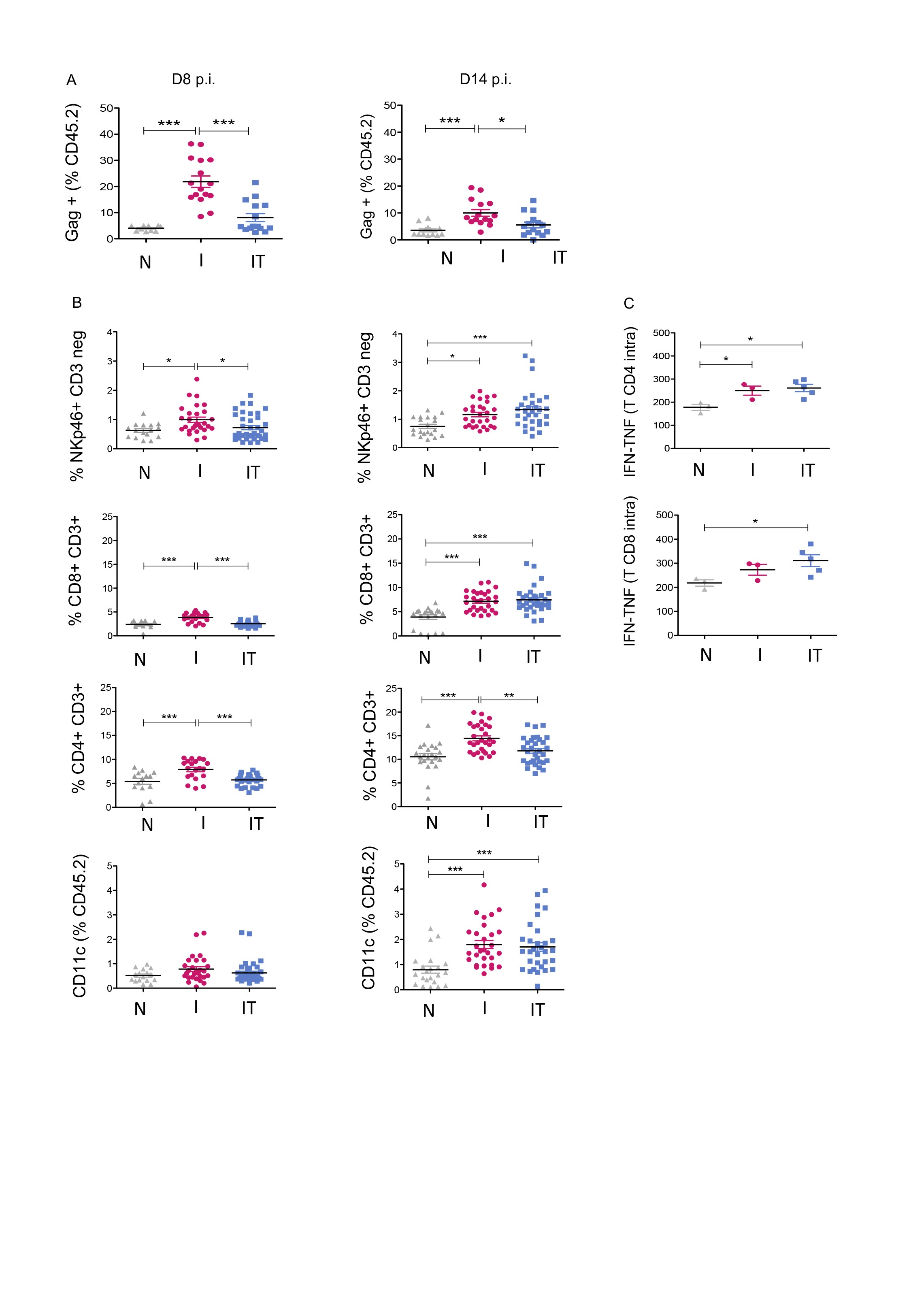

Supplement: Supplemental Material [file TEMI_A_1913068_SM2085.zip › Supplemental Figures/Suppl Fig 3.jpg]

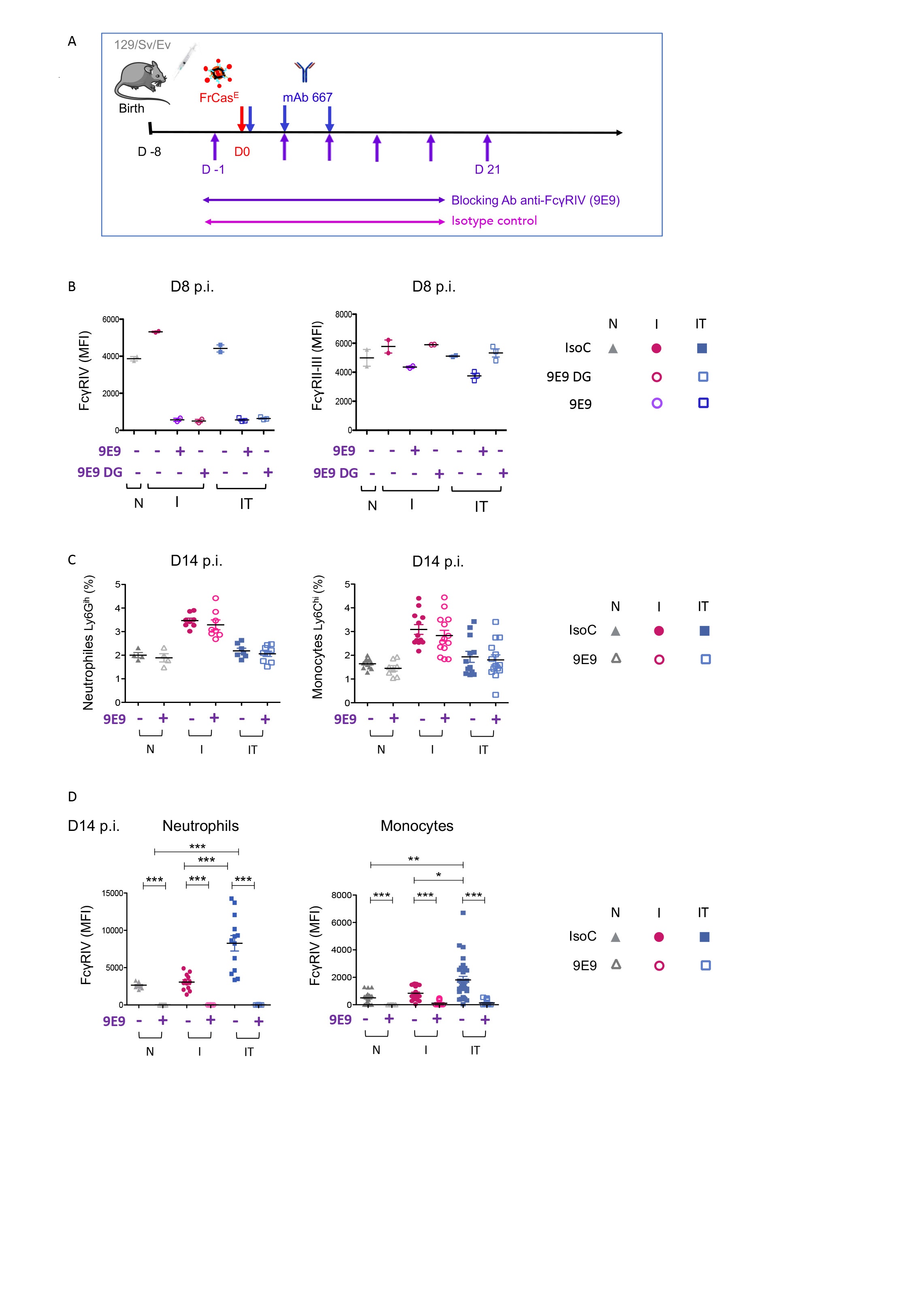

Supplement: Supplemental Material [file TEMI_A_1913068_SM2085.zip › Supplemental Figures/Suppl Fig 4.jpg]
