# Supplementary material for: Differential and sequential immunomodulatory role of neutrophils and Ly6Chi inflammatory monocytes during antiviral antibody therapy
Source: Emerg Microbes Infect. 2021 May 21;10(1):964–81. doi: 10.1080/22221751.2021.1913068 (PMC8158214; doi:10.1080/22221751.2021.1913068)
Supplement: Supplemental Material [file TEMI_A_1913068_SM2082.docx]

**SUPPLEMENTAL FIGURES**

***Supplemental Figure 1.* Functional activation of neutrophils stimulated with viral determinants or LPS.** BM-derived neutrophils were isolated from naïve mice and stimulated for 24h *in vitro* with viral determinants (virus or viral IC) or LPS (1µg/ml). **A**. *Cytokine secretion profile of neutrophils stimulated by virus (red), viral ICs (blue) or unstimulated (grey).* Cytokine release was assessed by using a flow cytometry-based multiparametric assay in supernatants of neutrophils isolated from BM of naive mice (>95% purity) and stimulated for 24 h. The data represent 5 independent experiments. Data are expressed as means +/- SEM. Statistical significance was established using a parametric 1-way ANOVA test (*p < 0.05; **p < 0.01; ***p < 0.001). **B-D**. *Functional activation of neutrophils stimulated by LPS*. Activation was assessed by monitoring the CD11b expression level and the frequency of CD11b^hi^ CD62L^lo^ neutrophils (**B**) as well as the cytokines (**C**) and chemokines (**D**) release monitored by using a flow cytometry-based multiparametric assay in supernatants of neutrophils isolated from BM of naive mice (>95% purity) and stimulated for 24 h by LPS (green) or left unstimulated (grey). The data represent 12 independent experiments. Data are expressed as means +/- SEM. Statistical significance was established using a paired Student’s *t* test (*p < 0.05; **p < 0.01; ***p < 0.001).

***Supplemental Figure 2:* Functional activation of monocytes stimulated with viral determinants or LPS.** BM-derived monocytes were isolated from naïve mice and stimulated for 24h *in vitro* with viral determinants (virus or viral IC) or LPS (1µg/ml). **A**. *Cytokine secretion profile of monocytes stimulated by virus (red) o viral ICs (blue) or unstimulated (grey).* Cytokine release was assessed by using a flow cytometry-based multiparametric assay in supernatants of monocytes isolated from BM of naive mice (>95% purity) and stimulated for 24 h. The data represent 6 independent experiments. Data are expressed as means +/- SEM. Statistical significance was established using a parametric 1-way ANOVA test (*p < 0.05; **p < 0.01; ***p < 0.001). **B-D**. *Functional activation of monocytes stimulated by LPS*. Activation was assessed by monitoring the CD80 and CD86 expression level (**B**) as well as the cytokines (**C**) and chemokines (**D**) release monitored by using a flow cytometry-based multiparametric assay in supernatants of monocytes isolated from BM of naive mice (>95% purity) and stimulated for 24 h by LPS (green) or left unstimulated (grey). The data represent 5 independent experiments. Data are expressed as means +/- SEM. Statistical significance was established using a paired Student’s *t* test (*p < 0.05; **p < 0.01; ***p < 0.001).

***Supplemental Figure 3*: Frequencies of infected splenocytes and immunological environment composition at day 8 (D8) and 14 (D14) p.i.** (**A**). *Frequencies of infected splenocytes.* Splenocytes from naïve (N), infected/non-treated (I), and infected treated (IT) mice were analyzed by flow cytometry on days 8 p.i. and 14 p.i. for retroviral positivity of splenocytes (percentage of Gag+ cells) gated in the CD45.2+ population. Data represent 3 independent experiments at D8 p.i. and 3 independent experiments at D14 p.i, with at least 4-5 mice (I and IT) per group and 2-3 mice for control groups. **B***. Immunological environment at D8 p.i. and D14 p.i*. Frequencies of NK cells, CD4^+^ and CD8^+^ T lymphocytes and CD11c^+^ cells were established by flow cytometry at D8 p.i. and D14 p.i. Data represent 4 independent experiments at D8 p.i. and 4 independent experiments at D14 p.i, with at least 4-5 mice (I and IT) per group and 2-3 mice for control groups. Data are expressed as means +/- SEM. Statistical significance was established using a parametric 1-way ANOVA test with Bonferroni’s multiple comparisons post-tests (*p < 0.05; **p < 0.01; ***p < 0.001). **C*.*** *Inflammatory cytokines production by T lymphocytes at D14 p.i*. Intracellular TNFα and IFNγ production by CD4^+^ and CD8^+^ T lymphocytes at D14 p.i. Data represent 1 experiment*.*

***Supplemental Figure 4: Efficiency and specificity of FcγRIV-blocking strategy.*** (**A**). Scheme of the *FcγRIV-blocking strategy***.** Eight-day-old 129/Sv/Ev mice were infected by intraperitoneal (i.p.) administration of 50 μl of a viral suspension containing 50,000 focus-forming units (FFU) and treated, or not, with 30 μg of 667 mAb, 1-hour p.i. and on days 2 and 5 p.i. by i.p. administration. In parallel, FcγRIV*-*blocking mAb (9E9) (full shapes) or an isotype control (IsoC) (open shapes) were administered at the dose of 5µg/g, every 3 days starting one day before the infection until day 21 p.i. (D21), i.e., the time necessary to eliminate the therapeutic 667 mAb. (**B**). *Specific FcγRIV-blocking mAb (9E9).* To ensure the specificity of the FcγRIV-blocking, the 9E9 mAb was deglycosylated to avoid interaction of its Fc Fragment with other FcγRs expressed on FcγRIV-expressing cells. The specificity was evaluated by measuring the expression of FcγRIV and FcγRIII on neutrophils surface at day 8 p.i. (D8) by flow cytometry. Data represent 1 experiment. (**C**). *FcγRIV-blocking does not affect the frequencies of neutrophils and monocytes in the spleen.* Frequencies of neutrophils and monocytes in the spleen of naive, infected/non-treated (I/NT), and infected treated (I/T) mice, in the presence or in the absence of FcγRIV-blocking mAb were analyzed by flow cytometry at day 14 p.i (D14) gated in the CD45.2+ population. **D**. *Efficacy of the FcγRIV-blocking*. FcγRIV expression was determined by flow cytometry at D14 p.i.. Data represent 3 independent experiments (**C**, **D**). Data are expressed as means +/- SEM. Statistical significance was established using a parametric 1-way ANOVA test with Bonferroni’s multiple comparisons post-tests (*p < 0.05; **p < 0.01; ***p < 0.001).
